# Association of modified cytosines and the methylated DNA-binding protein MeCP2 with distinctive structural domains of lampbrush chromatin

**DOI:** 10.1186/1756-8935-6-S1-P6

**Published:** 2013-03-18

**Authors:** Garry T Morgan, Peter L Jones, Michel Bellini

**Affiliations:** 1 Centre for Genetics and Genomics, School of Biology, University of Nottingham, Queens Medical Centre, Nottingham NG72UH, UK; 2 Boston Biomedical Research Institute, 64 Grove Street, Watertown, MA 02472, USA; 3 Department of Cell and Developmental Biology, University of Illinois, 601 S. Goodwin Avenue, Urbana, IL 61801, USA

## 

The lampbrush chromosomes (LBCs) of the amphibian oocyte are the largest known chromosomes and offer the unique opportunity to visualize the distinctive closed and open chromatin structural domains with an unprecedented spatial resolution. In this study we investigated the association of DNA methylation and proteins interpreting methylation state, such as MeCP2, with LBCs. We expressed HA-tagged MeCP2 in *Xenopus laevis* oocytes, and we observed that while it predominantly targeted the transcriptionally inactive chromomeres, a minor fraction of HA-MeCP2 also associated with many of the transcriptionally active lateral loops of LBCs. We further demonstrated that the association of MeCP2 with LBCs is directly determined by its 5-methylcytosine-binding domain. We then defined the distribution of 5-methylcytosines (5mC) by immunostaining *Xenopus* and axolotl LBCs and confirmed the pattern suggested by the expression of HA-MeCP2 targeting of intense staining of the chromomeres and of many loop bases. In addition, we found that short interstitial regions of the active transcriptional units could be clearly stained for 5mC. Interestingly, these 5mC-positive regions corresponded precisely to segments of active transcription units from which RNA polymerase II and nascent transcripts were simultaneously absent. Together, these data support a model presenting MeCP2 as both a transcriptional repressor and activator.

